# Xanthohumol Inhibits Notch Signaling and Induces Apoptosis in Hepatocellular Carcinoma

**DOI:** 10.1371/journal.pone.0127464

**Published:** 2015-05-26

**Authors:** Selvi Kunnimalaiyaan, Kevin M. Sokolowski, Mariappan Balamurugan, T. Clark Gamblin, Muthusamy Kunnimalaiyaan

**Affiliations:** Department of Surgery, Division of Surgical Oncology and Medical College of Wisconsin Cancer Center, Medical College of Wisconsin, Milwaukee, WI, United States of America; University of Medicine, Greifswald, Germany, GERMANY

## Abstract

Despite improvement in therapeutic strategies, median survival in advanced hepatocellular carcinoma (HCC) remains less than one year. Therefore, molecularly targeted compounds with less toxic profiles are needed. Xanthohumol (XN), a prenylated chalcone has been shown to have anti-proliferative effects in various cancers types in vitro. XN treatment in healthy mice and humans yielded favorable pharmacokinetics and bioavailability. Therefore, we determined to study the effects of XN and understand the mechanism of its action in HCC. The effects of XN on a panel of HCC cell lines were assessed for cell viability, colony forming ability, and cellular proliferation. Cell lysates were analyzed for pro-apoptotic (c-PARP and cleaved caspase-3) and anti-apoptotic markers (survivin, cyclin D1, and Mcl-1). XN concentrations of 5μM and above significantly reduced the cell viability, colony forming ability and also confluency of all four HCC cell lines studied. Furthermore, growth suppression due to apoptosis was evidenced by increased expression of pro-apoptotic and reduced expression of anti-apoptotic proteins. Importantly, XN treatment inhibited the Notch signaling pathway as evidenced by the decrease in the expression of Notch1 and HES-1 proteins. Ectopic expression of Notch1 in HCC cells reverses the anti-proliferative effect of XN as evidenced by reduced growth suppression compared to control. Taken together these results suggested that XN mediated growth suppression is appeared to be mediated by the inhibition of the Notch signaling pathway. Therefore, our findings warrants further studies on XN as a potential agent for the treatment for HCC.

## Introduction

Hepatocellular carcinoma (HCC) is the third leading cause of cancer-related deaths globally [1]. Approximately 70% of patients present with advanced disease often with concomitant cirrhosis. Consequently, the 5-year survival for these patients is 50–70%[1]. Currently, the single effective curative modality is surgical resection; however, given the metastatic potential and comorbidities surrounding patients with HCC, surgery is often non-efficacious. As a result, palliative care is often the mainstay of treatment strategies. Sorafenib, a multi-kinase inhibitor, is the only Food and Drug Administration approved systemic therapy. However, sorafenib has a limited survival advantage of approximately 11 weeks and is effective in nearly one-third of patients [2, 3]. Given the increasing understanding of signaling pathways and the limited treatment options to date, the development of new therapeutic strategies is integral [4, 5].

Over-expression of Notch receptors and their ligands were detected in HCC tumor tissues and cell lines compared to normal liver [4, 6–8]. Importantly, inhibition of Notch1 in HCC cells by shRNA against Notch1 or gamma secretase inhibitors resulted in cell cycle arrest or apoptosis [9–13]. Recently, aberrant expression of Notch1 has been correlated with HCC metastasis and inhibition of Notch1 prevented metastasis both in vitro and in vivo [6, 14]. Therefore, inhibition of the Notch1 signaling pathway could be a promising target for new anticancer therapeutic drugs. In this regard, gamma secretase inhibitors (GSI), inhibition of the Notch transcription complex, and the development of antibodies targeting specific Notch receptors and ligands have shown great potential as new targeted therapeutic agents [13, 15–17].

One particular area of interest is the use of natural products such as flavonoids as they exhibit targeted therapeutic options by altering various signaling pathways. Their effectiveness as anti-inflammatory, anti-oxidant, and anti-angiogenic agents are well documented. In addition, their high bioavailability and limited toxicity profiles provide them as ideal candidates in chronically ill patients. Despite this, their anti-tumorigenic effectiveness has enriched their use as a potential cancer strategy. Xanthohumol (XN), a natural phytochemical isolated from the cones of hop plant (*Humulus lupulus* L.) has demonstrated inhibition of cancer cell proliferation in vitro in several solid organ-specific tumors such as breast, colon, hepatocellular, medullary thyroid, ovarian, pancreatic, and prostate [18–27]. XN attenuates cellular growth through the induction of both caspase-dependent and independent apoptosis [24, 28–30]. Translating to an in vivo model, XN administration tempered tumor progression in advanced stage disease of the prostate [26]. In addition to its promising anti-tumorigenic ability, XN has shown to have a low toxicity profile as well as high bioavailability. Recent in vivo studies revealed that orally administered XN resulted in both small and large intestinal absorption and that it did not affect major organ function including the female reproductive system [22, 31–33]. Despite the early promising findings in the various malignancies, there is insufficiency in a well-accepted mechanism by which XN mitigates carcinogenesis.

In the present study, we examined the anti-proliferative effects of XN on established human HCC cell lines. We provide evidence that XN inhibited cellular growth and that XN-treatment induced apoptosis as well as inhibited Notch signaling. Ectopic expression of Notch1 reversed XN-induced suppression in HCC cells. These findings suggest that the mechanism by which HCC cellular proliferation is reduced following XN treatment appears to be mediated by the inhibition of the Notch signaling pathway.

## Materials and Methods

### Cell lines and culture conditions

The human hepatocellular carcinoma (HCC) cell lines (HepG2, Hep3B, and SK-Hep-1) were purchased from American Type Culture Collection (ATCC, Rockville, MD, USA) and Huh-7 cells were a kind gift from Dr. Chisari, The Scripps Research Institute, La Jolla, CA. HCC cell lines (HepG2, Hep3B, and SK-Hep-1) were cultured in Eagle's Minimum Essential Medium (EMEM) whereas Huh-7 was cultured in Dulbecco's Modified Eagle's Medium (DMEM) supplemented with 10% fetal bovine serum (FBS) and 1% penicillin/streptomycin (all were from Invitrogen, Carlsbad, CA, USA) at 37°C in a humidified atmosphere containing 5% CO_2_. Huh-7 cells were further supplemented with nonessential amino acids (NEAA, Life Technologies, Carlsbad, CA, USA) and 4-(2-hydroxyethyl)-1-piperazineethanesulfonic acid (HEPES, Life Technologies). The culture media was replaced every 2–3 days. The confluent cells were sub-cultured by splitting them at 1:5 ratios.

### Reagents and treatment

Xanthohumol (XN), 3-(4, 5-dimethylthiazol-2-yl)-2, 5-diphenyltetrazolium bromide (MTT), and dimethyl sulfoxide (DMSO) were purchased from Sigma-Aldrich (St. Louis, MO, USA). XN was dissolved in DMSO into stock concentrations of 50mM. Antibodies against survivin, glyceraldehyde phosphate dehydrogenase (GAPDH), Notch1 (epitope from C-terminus), HES-1, cyclin D1, Bcl2, Mcl1, total PARP, and caspase-3 were purchased from Santa Cruz Biotechnology (Santa Cruz, CA, USA) and cleaved caspase-3 and cleaved PARP were obtained from Cell Signaling Technology Inc. (Danvers, MA, USA) respectively.

### Cellular proliferation, viability, and colony forming assays

Cellular proliferation and viability on the panel of HCC cell lines was measured by using colorimetric assay with MTT. Cells were seeded in 24-well plates and allowed to adhere overnight. Cells were then treated with indicated concentrations (0–15μM) of XN in quadruplicates. Treated cells were maintained in a time course fashion up to 96 hours. Viability was assessed by replacing media with 250μL of Roswell Park Memorial Institute media (RPMI, Life Technologies) containing 0.5mg/mL MTT and incubated for three hours. Following incubation, 750μL DMSO was added to dissolve the insoluble formazan to produce a colored solution with which the absorbance was measured at 540nm using a spectrophotometer (Infinite M200 PRO; TECAN, San Jose, CA, USA). The reported results represent the average of three experiments. The effect of XN on colony forming ability was determined by the measurement of the colonogenic cell survival as previously described [34].

### Real time non-invasive cellular proliferation assay

Determination of cellular proliferation during real time was achieved through using IncuCyte Live-Cell Imaging Systems (Essen Bioscience, Ann Arbor, MI, USA). Huh-7 and Hep3B cells (3–5 X10^3^ cells) were seeded onto a 96-well plate and incubated in an XL-3 incubation chamber maintained at 37°C. At 12 hours, cells were treated with varying concentrations (0–50μM) of XN up to 96 hours. Cells were imaged every two hours using 10X objective for the duration of the experiment. Cellular confluence was calculated using IncuCyte 2011A software. Cellular proliferation was expressed as an increase in confluence as a percentage at 12-hour intervals.

### Western blot analysis

After 96 hours of XN treatment, cells were collected and lysed in radioimmunoprecipitation assay (RIPA, Thermo Fisher Scientific, Waltham, MA, USA) buffer supplemented with a protease inhibitor cocktail (Sigma-Aldrich) and phenylmethylsulfonylflouride (PMSF, Sigma-Aldrich). Protein concentrations were quantified using the bicinchonic acid assay method (BCA, Thermo Fisher) and analyzed by SDS-PAGE (Bio-Rad Laboratories, Hercules, CA, USA). Protein was then transferred to nitrocellulose membranes (Bio-Rad Laboratories) using a Trans-Blot Turbo (Bio-Rad Laboratories). Following transfer, membranes were blocked in 5% milk solution for one hour. After blocking, the membranes were incubated overnight at 4°C with their respective primary antibodies. The next day, membranes were washed with PBS-T wash buffer (1X PBS, 0.05% Tween-20) three times for five minutes each. Membranes were then incubated for a minimum of 1.5 hours in either anti-rabbit or anti-mouse secondary antibody (Santa Cruz Biotechnology, 1:15,000 dilution). Following secondary antibody incubation, membranes were washed again three times for five minutes each. Detection of immune complexes was assessed using chemiluminescence with a HRP antibody detection kit (Femto super signal, Dura super signal (Thermo Fisher), Clarity (Bio-Rad Laboratories)). Images of the complexes were taken using the Molecular Imager ChemiDoc XRS^+^ imager with image software (Bio-Rad Laboratories).

### Caspase-3 and -7 activities

Further apoptotic studies measured the cleavage of caspase-3 and -7 using the Caspase-Glo 3/7 Assay (Promega, Madison, WI, USA) from XN-treated cellular lysates. Ten to fifteen μg of protein samples in 25μL total volume was mixed with equal volume of Caspase-Glo reagent and incubated at room temperature in a white, 96-well plate for thirty minutes. Activity level of caspase family was determined by the measurement of luminescence using the Infinite M200PRO Microplate reader (TECAN).

### Notch1 depletion

To determine the effect of Notch1 depletion, shRNA against Notch1 (two different sequences) containing plasmids (Sigma-Aldrich) were transfected into Huh-7 cells for 48 hr using Lipofectamine 2000 (Invitrogen) reagent in OPTIMEM media (Invitrogen) as per the manufacturer’s protocol. As a control, a nonspecific, no-target sequence containing shRNA plasmid was used. Then cell lysates were prepared for use in further experiments.

### Statistical analysis

Determination of statistical significance was calculated by analysis of variance (ANOVA) using a statistical analysis software package (IBM SPSS Statistics version 22, New York, NY, USA). Statistical significance was achieved with p-values of < 0.05. Data were represented as ± standard error.

## Results

### Xanthohumol reduces HCC cellular viability and colony forming ability

**Fig 1:**
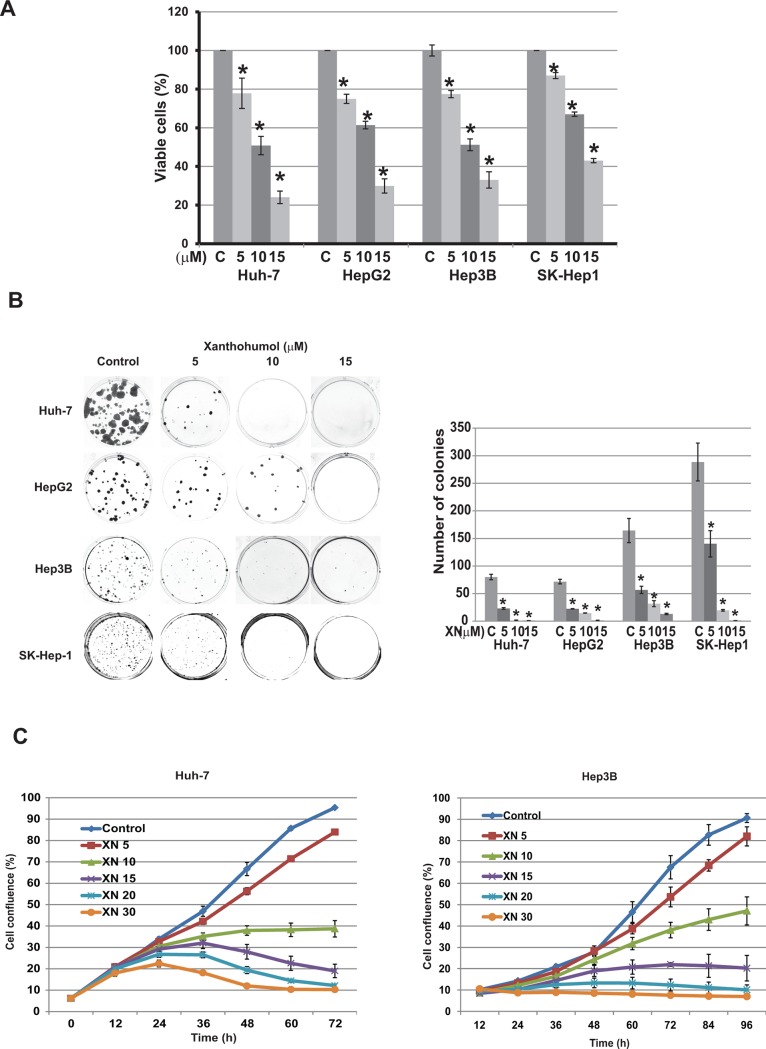
Effect of XN on human hepatocellular carcinoma cellular proliferation. A. Commonly used four human hepatocellular carcinoma cancer cell lines were treated with XN at indicated doses for 4 days and cytotoxicity was measured using MTT assay (n = 3; p>0.05 at 5 μM and above concentrations for all cancer cell lines compared to control treatment. B. Cells were treated with XN up to 15 μM for 4 days and cell viability was measured by colony formation assay and the number of colonies were counted and showed in bar graph. (p>0.05 at 5 μM and above concentrations compared to control treatment. C. Effects of XN on Huh-7 and Hep3B cell proliferation in real-time. Cells were treated with indicated concentrations of XN and cell proliferation was monitored in real time with the continuous presence of XN. The cells were photographed and the cell confluence was calculated using IncuCyte 2011A software. The changes in cell confluence are used as a surrogate marker of cell proliferation. Statistically significant (p<0.05) growth suppressions were observed at or above 10 μM XN compared to control.

The effect of Xanthohumol (XN) on HCC cellular viability of four established HCC cell lines (Huh-7, HepG2, Hep3B, and SK-Hep-1) was evaluated by MTT assay. As shown in Fig 1A, treatment of Huh-7, HepG2, Hep3B, and SK-Hep-1 cells with varying (0–15μM) XN concentrations for 96 hours resulted in a dose-dependent reduction in cellular viability compared to control (DMSO). To further confirm the cell viability after treatment with XN, colonogenic assay was performed. HCC cells treated at or above 5μM XN, showed significant loss of colony forming ability (Fig 1B). This was further confirmed by live cell imaging system at increasing concentrations (0–50μM) of XN treatment in Hep3B and Huh-7 cells. Cellular proliferation measured by increase in cell confluency via IncuCyte Live-Cell Imaging is shown in Fig 1C. Compared to control (DMSO), cellular proliferation was significantly decreased as the concentrations of XN increased.

### Xanthohumol promotes apoptotic induction in hepatocellular cancer cells

**Fig 2:**
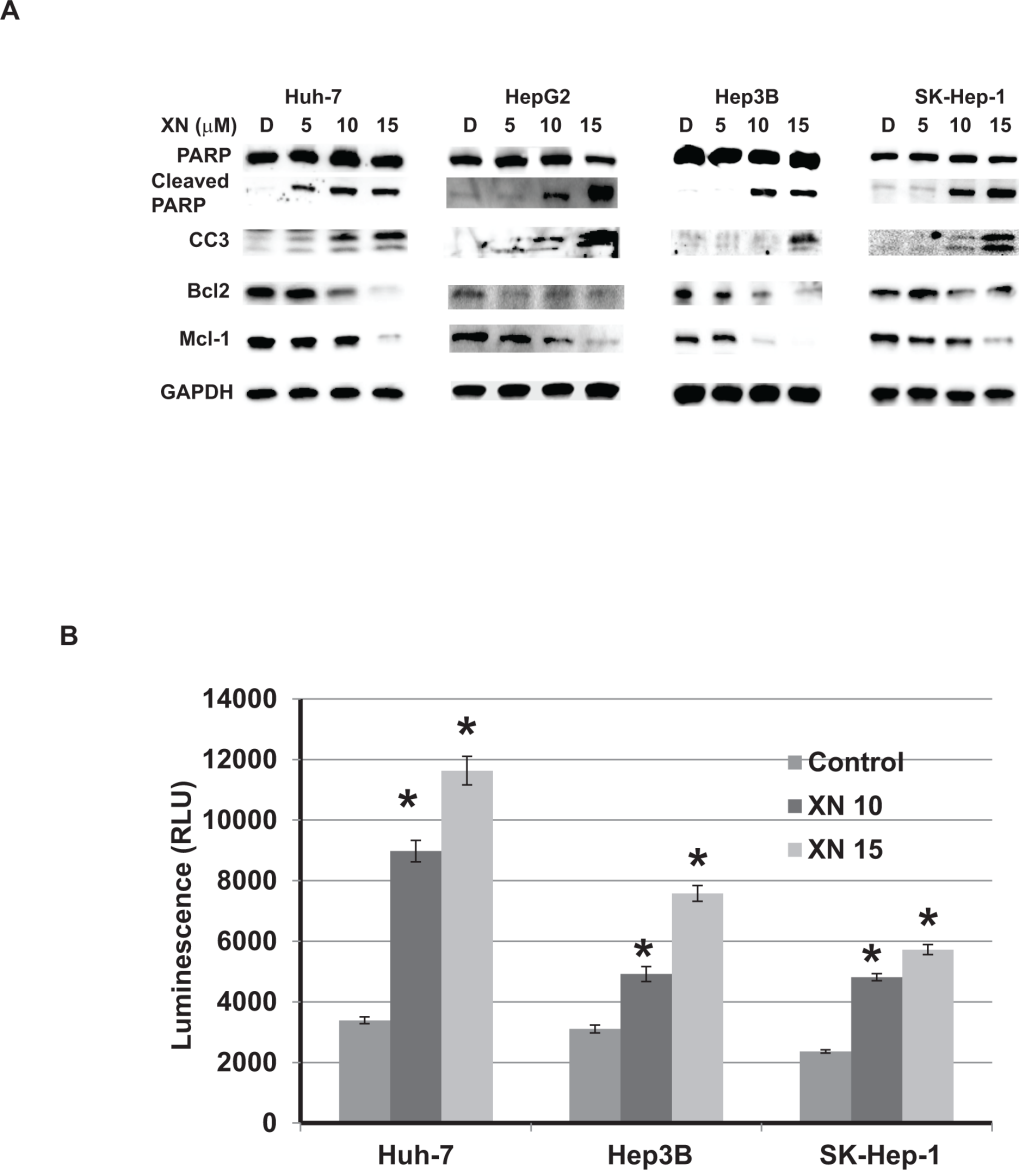
Mechanism of action of XN in hepatocellular carcinoma cell lines. A. Levels of cleaved PARP, and cleaved caspase-3 (markers of apoptosis) were analyzed from lysates after 48hrs of XN treatment by Western. A level of anti-apoptotic protein Bcl2 and Mcl-1 were also determined. GAPDH was used as loading control. B. Caspase-3 and -7 activities were measured by caspase-Glo3/7 assay. (n = 3; p<0.05 compared to control in all cell lines tested).

To investigate the inhibitory effect of HCC proliferation in XN-treated cells, first we analyzed apoptotic markers by Western analysis. As shown in Fig 2A, XN induced cleavage of pro-apoptotic markers such as PARP as well as caspase-3. Correlating with this, there is a reduction of anti-apoptotic markers, Bcl-2 and Mcl-1 with increasing concentrations of XN. These results were confirmed by luminescence assay measuring caspase-3 and -7 activity levels. Fig 2B shows an increase is caspase activity in cell lysates from XN-treated cells. These results suggest that XN inhibits cellular proliferation by inducing apoptosis in HCC in vitro.

### Xanthohumol inhibits Notch1 signaling in hepatocellular carcinoma

**Fig 3:**
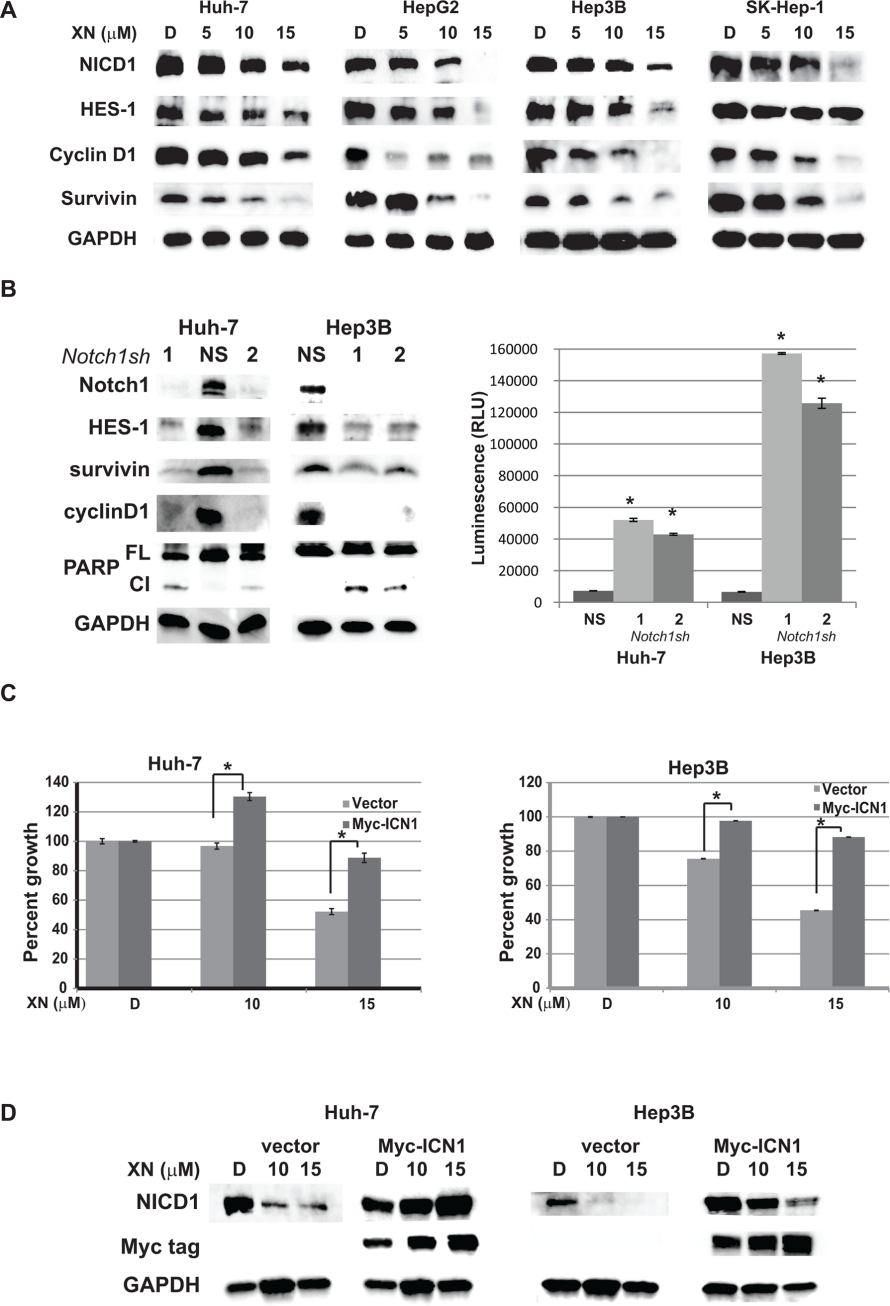
XN inhibits Notch1 signaling pathway. A. Protein levels of intracellular domain (NICD), HES-1, an immediate downstream target of Notch1 signaling, cyclinD1, and survivin were analyzed by western blot after XN-treatment. Equal loading was confirmed by GAPDH. B. Notch1 inhibition by shRNA (seq #1 and seq #2) induced apoptosis similar to XN treatment. Functional Caspase assay confirmed the induction of apoptosis in Notch1 knock-down cell lines. A non-specific, no target, scrambled sequence (NS) as control was used. GAPDH was used as gel loading control. C. Effect of active Notch1 overexpression in XN treatment. Huh-7 and Hep3B cells were transfected with Notch1plasmid (pcDNA-Myc-ICN1) and treated with or without XN. Percent of growth was measured by MTT assay (n = 3; p = 0.05 compared to vector with XN10). D. Western analysis of (pcDNA-Myc-ICN1) or empty vector transfected Huh-7 and Hep3B cells treated with or without XN for the expression of the exogenous Notch1 protein. Myc-tag antibody was used to determine the level of exogenous Notch expression.

XN has shown to reduce Notch1 expression in ovarian cancer cells but the mechanistic action of XN is not clear [24]. Therefore, we sought to analyze the mechanism of action by which XN inhibits HCC cell growth and the role of the Notch1 signaling pathway in XN-treated cells. We measured the protein levels of the Notch signaling pathway members via Western analysis after treatment with XN. As seen in Fig 3A, active Notch1 is dramatically reduced in all HCC cell lines tested. This reduction in Notch1 protein is associated with reduction of its downstream targets such as HES-1, cyclin D1, and survivin proteins. To confirm if HES-1, cyclin D1, and survivin proteins reduction is associated with Notch reduction, Huh-7 and Hep3B cells were transfected with shRNA against Notch1 as well as a control, no target, nonspecific sequence separately for two days and cell lysates were analyzed. As shown in Fig 3B, knockdown of Notch1 reduced HES-1, survivin, and cyclin D1 proteins. Additionally, depletion of Notch1 following transfection initiated apoptosis shown by an increase in the cleaved PARP protein. Collectively, these results suggest that XN inhibits HCC cell growth and the Notch signaling pathway.

### Over-expression of active Notch1 reverses the growth suppression effect of XN in hepatocellular carcinoma

The results from our study demonstrated that Notch signaling suppression may be essential for the documented anti-proliferative effect of XN in HCC. To determine if Notch1 pathway mitigation mediates the suppressive effect of XN, we over-expressed either active Notch1 tagged with Myc (Myc-ICN1) or empty vector in Huh-7 and Hep3B cells, then treated with XN for 2 days, and measured cell viability. The plasmid pcDNA3-myc-ICN1 and pcDNA3 were a kind gift of Dr. Jon Aster, Boston, MA. When Notch1 is over-expressed, XN treatment showed less growth suppression as compared to cells transfected with empty vector (Fig 3C). However, the reduction observed in empty vector with XN treatment is lower than the cells treated with XN as shown in Fig 1. The expression of transfected Notch1 was confirmed by western analysis (Fig 3D). However, these results of NICD1 over-expression rescuing XN inhibitory effects indicate that inhibition of Notch1 signaling is important for the growth suppression effect of XN.

## Discussion

HCC remains a highly aggressive and difficult to treat cancer in the presence of concomitant cirrhosis and chemoresistance. In advanced disease, which represents a large cohort, systemic therapy is indicated; however, current treatment modalities are limited in scope and effectiveness. Given the limited therapeutic options, the need for targeted molecular therapy is a foremost necessity. Moreover, natural products have had early success both as single and as multiple combination therapies in several cancer types. Xanthohumol, a phytochemical isolated from the hop plant, has significant anti-tumor activities against breast, colon, HCC, medullary thyroid, ovarian, pancreatic, and prostate cells [18, 19, 21–27, 30, 35]. Though similar growth suppression effect is observed in variety of cancers, including HCC, the associated effects are correlative; it has been shown to induce both caspase dependent and caspase independent apoptosis, inhibit cell invasion, angiogenesis, nuclear factor activation, and Notch1 reduction [25, 28–30, 36, 37]. Despite these studies, the molecular mechanism of growth suppression remains unclear.

In this current study, we have shown that XN inhibits growth of HCC cells by apoptosis in a dose-dependent manner. The decrease in cellular growth and resultant induction of apoptosis is mechanistically driven by down regulation of Notch1 signaling supported by the over-expression of active Notch1 negating XN effects. For the first time, we have presented here that XN induces growth suppression in HCC cells which directly involves the Notch signaling pathway.

It has been reported that cyclin D1 and survivin are downstream targets of Notch1 and collectively play a role in chemoresistance in cancer [9, 38–40]. Here we have shown that XN decreases growth in HCC cells and inhibits Notch signaling pathway and its immediate downstream targets. We therefore predict that inhibition of Notch signaling may sensitize HCC cells to XN by preventing pro-survival, chemoresistant-like proteins such as cyclin D1 and survivin.

As previously mentioned, XN has been shown to exhibit high bioavailability and with a limited toxicity profile [22, 31, 32, 36]. In addition to the favorable pharmacodynamics, XN also demonstrated to reduce the growth of poorly differentiated prostate tumors all without adverse side effects. Moreover, a metabolism and pharmacokinetic clinical trial conducted in healthy men and women following oral consumption of XN demonstrated limited side effects and high bioavailability [41]. This in vivo data along with what we provide suggest that XN may be a novel agent for the management of multiple solid organ tumors, including HCC. In conclusion, Xanthohumol represents a promising, safe, and highly effective natural product against hepatocellular carcinoma in vitro. Future work should further assess XN effectiveness in vitro with combination studies including the FDA-approved sorafenib as well as other chemotherapy agents.
